# Real‐time detection of agitation in people with dementia: The RadioMe system

**DOI:** 10.1002/alz70863_110644

**Published:** 2025-12-23

**Authors:** Nicolas Farina, Stephen Brewster, Patrizia Di Campli San Vito, Jorg Fachner, Alex Street, Eduardo Miranda, Sube Banerjee

**Affiliations:** ^1^ University of Plymouth, Plymouth UK; ^2^ University of Glasgow, Glasgow UK; ^3^ Anglia Ruskin University, Cambridge UK; ^4^ University of Nottingham, Nottingham UK

## Abstract

**Background:**

Agitation is a common comorbidity of people with dementia and can be particularly distressing. Technology may be a valid way to detect agitation in people with dementia in real time to allow for intervention. RadioMe combines agitation detection technology (e.g. through wearables) alongside relaxing music response to support people to live independently within the community. The system seamlessly mixes music with a live radio and can incorporate diary reminders too.

**Method:**

The RadioMe system was installed in 20 community‐dwelling people with dementia with agitation symptoms for up to four months, in this proof‐of‐concept study. In the first two months participants had access to the radio and diary reminder elements of the system; in the second two months participants also had access to the relaxing music playlist elements. We measured quality of life, agitation and implementation outcomes across the timepoints. Qualitative interviews were used to better understand participants’ experiences of the system.

**Result:**

Findings indicate that people with dementia were generally satisfied with the system (Mdn = 3.5), and perceived it to be acceptable (AIM, Mdn =3.7), appropriate (Mdn = 3.5) and feasible (Mdn = 3.5). There was evidence that participants did struggle to integrate it into their day‐to‐day lives, and there were frequent issues with the setup and connectivity of the wearables. There was no evidence that the system had a detrimental effect on people's quality of life or agitation symptoms.

**Conclusion:**

An automated system to detect agitation and respond with relaxing music has potential value, but ensuring the technology is accessible and not disruptive should be a priority before it can be adopted more widely for people with dementia.

